# Loneliness among older adults who have experienced homelessness: qualitative findings from the HOPE HOME study

**DOI:** 10.21203/rs.3.rs-3517416/v1

**Published:** 2023-11-13

**Authors:** Yeqing Yuan, Kelly R. Knight, John Weeks, Stephen King, Pamela Olsen, Margot Kushel

**Affiliations:** School of Social Work, University of Alaska Anchorage; Department of Humanities and Social Sciences, School of Medicine, University of California - San Francisco; Center for Vulnerable Populations at Zuckerberg San Francisco General Hospital and Trauma Center, Department of Medicine, School of Medicine, University of California -San Francisco; Center for Vulnerable Populations at Zuckerberg San Francisco General Hospital and Trauma Center, Department of Medicine, School of Medicine, University of California -San Francisco; Center for Vulnerable Populations at Zuckerberg San Francisco General Hospital and Trauma Center, Department of Medicine, School of Medicine, University of California -San Francisco; Center for Vulnerable Populations at Zuckerberg San Francisco General Hospital and Trauma Center, Department of Medicine, School of Medicine, University of California -San Francisco

## Abstract

**Background:**

Loneliness is more common in older adults and those who face structural vulnerabilities, including homelessness. The homeless population is aging. We know little about loneliness among older adults who have experienced homelessness. We aimed to describe the loneliness experience among older adults who have experienced homelessness and the individual, social, and structural conditions that shaped these loneliness experiences.

**Methods:**

We purposively sampled 22 older adults from the HOPE HOME study, a longitudinal cohort study among adults aged 50 years or older experiencing homelessness in Oakland, California. We conducted in-depth interviews about participants’ perceived social support and social isolation. We conducted qualitative content analysis.

**Results:**

Twenty participants discussed loneliness experience, who had a median age of 57 and were mostly Black (80%) and men (65%). We developed a typology of participants’ loneliness experience and explored the individual, social, and structural conditions under which each loneliness experience occurred. We categorized the loneliness experience into four groups: 1) “lonely – distressed”, characterized by physical impairment and severe isolation; 2) “lonely – rather be isolated”, reflecting deliberate social isolation as a result of trauma, marginalization and aging-related resignation; 3) “lonely – transient”, as a result of aging, acceptance and grieving; and 4) “not lonely” – characterized by stability and connection despite having experienced homelessness.

**Conclusions:**

Loneliness is a complex and heterogenous social phenomenon, with older adults who have experienced homelessness exhibiting diverse loneliness experiences based on their individual life circumstances and needs. While the most distressing loneliness experience occurred among those with physical impairment and mobility challenges, social and structural factors such as interpersonal and structural violence during homelessness shaped these experiences.

## Introduction

Whereas social isolation is an objective condition characterized by a lack of supportive social network, loneliness is a subjective state of social isolation [1]. Loneliness is common among older adults; an estimated 3–43% of adults aged 65 years or older reported experiencing loneliness worldwide [2, 3]. Loneliness is associated with negative health consequences among older adults, including hypertension, cardiovascular disease, depression, and premature mortality [4, 5, 6, 7].

Individuals who have experienced structural vulnerabilities, including homelessness, may face greater challenges in forming social connections for several reasons. Individuals experiencing homelessness may have a smaller social support system prior to entry into homelessness, as research consistently showed that social isolation and loss of social support are primary contributors of homelessness [8]. Many individuals experiencing homelessness lack the material means to form and maintain social relationships (e.g., stable address, transportation, phone, money). The stigma associated with homelessness can lead to rejection and withdrawals from existing social relationships [9, 10]. Systemic and structural discrimination, such as criminalizing homelessness can lead to disruption of social networks.

The homeless population is considered “old” at age 50 due to their poor health and shortened life expectancy. Approximately half of the homeless population are 50 years or older, and 40% of older adults who have experienced homelessness reported experiencing loneliness [11].

Little is known about how loneliness experience may be shaped by the intersecting vulnerabilities of aging, homelessness, and social isolation. Investigating this question will expand the existing understanding of loneliness as a complex public health problem and inform targeted programmatic services and policies aimed at improving health and mental health outcomes among people experiencing or at risk for homelessness. In this qualitative analysis, we aimed to answer the following research questions: 1) How do older adults who have experienced homelessness experience loneliness; and 2) What individual (e.g., physical impairment), social (e.g., social isolation), and structural (e.g., homelessness) conditions shaped these loneliness experiences?

## Methods

### Study Design

This study used qualitative data collected as part of a longitudinal mixed-methods study. The parent study, Health Outcomes of People Experiencing Homelessness in Older Middle Age (HOPE HOME), aimed to examine the intersection between social isolation, functional and cognitive impairment, and use of supportive services among a cohort of older adults experiencing homelessness in Oakland, California. Using a population-based, multi-stage sampling design, HOPE HOME recruited a probability sample of 350 individuals from July 2013 to June 2014 and an additional 100 individuals from August 2017 to July 2018. Participants were eligible for the parent study if they met the following criteria: at least 50 years old, English speaking, consent to participate, and was homeless at recruitment, as defined by the federal Homeless Emergency Assistance and Rapid Transitions to Housing (HEARTH) Act (2010) [12]. Every six months, participants attended study visits in which staff conducted structured interviews and clinical assessments.

Between September 2018 and January 2019, we conducted semi-structured interviews with a purposive subsample of 22 participants who reported either one or more activities of daily living (ADL) limitations (e.g., dressing, bathing, eating), 2 or more instrumental activities of daily living (e.g., transportation, cleaning, managing finances), or had scores consistent with cognitive impairment on the 3MS (the modified mini-mental state test), while still having capacity to consent [13]. We recruited the sample purposively by social isolation and social support, so that half of the sample reported above average social support and below average levels of social isolation, and half reported below average social support and above average levels of social isolation. The interviewer team consisted of two Black men (JW and SK) and one White woman (PO) who received training on qualitative data collection. We recorded and transcribed interviews verbatim. All participants gave informed consent and the University of California, San Francisco’s Institutional Review Board approved all study protocols. Each participant received a $25 gift certificate to compensate for their time.

The interviews took place at a social service agency in Oakland, California from which HOPE HOME rents space as a research fieldsite. Interviews lasted approximately 60 minutes. Interviews started with a general question about participants’ current well-being status (e.g., how are you doing today?), followed by questions focusing on perceived social support and social isolation, experiences living with functional and/or cognitive impairment, strategies used to optimize function, assistance from caregivers, and experiences of receiving caregiving.

### Data Analysis

In this analysis, we included only the participants who discussed their loneliness experience. We conducted data analysis using a method consistent with qualitative content analysis [14]. This method aims to examine the mechanisms of a social phenomenon and its conditions by following four stages: familiarization, coding and category formation, data extraction, and data interpretation.

Familiarization with data entailed reading and re-reading the entire set of transcripts to understand the overall life experiences of each participant. While doing this, we summarized each transcript in the form of an “episode profiling” memo, which entailed creating an analytic summary of participants’ life course experiences [15].

We noticed variations in participants’ loneliness experiences as we became more immersed in the data. This prompted us to systematically examine the variation by comparing data responding to two specific interview questions related to loneliness: 1) have you felt lonely in the last seven days; and 2) what do you do when you feel lonely? We constantly compared these responses and interpreted them in the context of participants’ overall life experiences. Based on this data, we developed a typology of loneliness experience consisting of four categories and a list of codes for the remaining data based on the familiarization process. We conducted this step using a combination of a qualitative data management software (dedoose.com) and Microsoft Word.

Next, we re-arranged the entire data into four charts based on the loneliness categories described above, each representing one loneliness type. Each chart is a matrix in which each column is a data source (i.e., participant ID) and each row is a particular code (e.g., medical and functioning status). The cells of the matrix contain a summary of relevant data or highlighted quotes.

We interpreted the extracted data through two steps: pattern recognition and pattern integration. First, we further consolidated the extracted data to capture the most distinct feature of each loneliness group (i.e., pattern). This was done by making constant comparisons of cases within and across charts and removing redundant patterns or merging repetitive patterns. We paid attention to cases that did not fit the pattern and explored plausible reasons. Next, we made a conceptual connection between the loneliness typology and their respective group features in an attempt to describe the condition under which each loneliness experience took place. Data analysis concluded with the identification of unique characteristics or conditions for each loneliness group that are both empirical-based and conceptually relevant.

### Rigor

#### Analytic process.

We enhanced the analytical rigor by engaging in constant comparison of the data in search of confirming and disconfirming cases related to our pattern recognition. We conducted coding and data extraction in conjunction with memos to help us understand data in the context of participants’ overall experiences. While the first author (YY) took the lead in data analysis, she engaged in regular consultation with the co-author (KRK), the co-investigator of the parent study and a qualitative methods expert with substantive expertise in homelessness research, to enhance the soundness of the methods and validity of results.

#### Author reflexivity.

The first author, who led the data analysis, is a person of color who has several years of social work experience working with individuals experiencing homelessness on the streets or in shelters, often including older homeless adults. Her experience interacting with different types of homelessness services and healthcare systems, along with her research experience, enabled her to empathetically understand participants’ life experiences and pay close attention to how structural vulnerabilities may trigger or sustain the experience of loneliness.

## Results

### Participant characteristics

We included twenty participants who discussed their loneliness experience. The median age was 57, with a range of 50–66. The majority were Black (80%) and men (65%). All participants met the federal definition of homelessness when first recruited into the parent study. Seven participants were experiencing homelessness at the time of the interview, among whom 4 were unsheltered and 3 were sheltered.

### Loneliness: experiences and conditions

We developed a typology of participants’ loneliness experience and explored the individual, social, and structural conditions under which each loneliness experience was enacted or sustained. The four types of loneliness experiences were: 1) “lonely – distressed”, characterized by physical impairment and severe isolation; 2) “lonely – rather be isolated”, reflecting deliberate social isolation as a result of trauma, marginalization and aging-related resignation; 3) “lonely – transient”, as a result of aging, acceptance and grieving; and 4) “not lonely” – characterized by stability and connection despite having experienced homelessness. Collectively, these themes revealed a wide range of loneliness experiences among older adults who have experienced homelessness and the structural stressors and social marginalization that increased vulnerability to loneliness.

### Lonely – distressed: impairment and isolation

Participants in this group explicitly stated that they were currently experiencing loneliness, and many expressed a strong desire for companionship: *“[I would like] just the companionship, someone to dialog with, someone to just have a conversation with.”* When asked whether he is lonely, a participant aid, *“Oh, man, all the time. That’s thorough with me”*. The extent of loneliness was reflected in participants’ distressing coping behaviors, such as heavy drinking or frequent crying. As one participant described, when he felt lonely, he drank hard liquor and went to sleep to *“cover it up.”*

The most distinct feature of this group is significant physical impairment. Among the numerous medical and physical complications, almost all participants in this group reported having sustained injuries. These injuries had been inadequately treated, leaving participants with chronic pain and mobility challenges, as one participant described: *“I’m in so much pain, it’s hard for me to get out the bed sometimes. I finally got – I worked out a way to get my clothes on, but sometimes it’s just hard to do anything.”* The constant physical discomfort brought about mental distress or exacerbated ongoing depression, as one participant explained: *“[I]t’s nerve pain. And when it hits, I gotta worry, is that gonna make my muscle cramp, is that sending a signal, it’s gonna cause a problem, will I be able to walk? All of that. Goes through my mind. So I have to constantly struggle through that mentally. And I have to constantly be aware of the do’s and don’t’s. And that makes me feel inadequate.”*

Participants in this group reported difficulty forming or maintaining social support. On one hand, participants reported that the severe physical impairment made it challenging to engage in social activities, posing barriers to forming and maintaining new social support systems. This was true not only for those with impaired mobility but also for participants with sensory impairment. For example, one participant who had hearing loss reported: *“Most people don’t like when you want them to repeat themselves. So I basically stay out of [the] subject, you know, conversations. It really messes up my social life.”* On the other hand, participants described the limited and fragile nature of their existing social networks. Most participants’ small social networks were available only for instrumental support such as assisting with household chores. One participant had to cut off ties with someone he used to hang out with for emotional support because of their maladaptive coping behaviors (e.g., substance use), which was interfering negatively with the participant’s own recovery.

### Lonely – rather be isolated: trauma and resignation

Participants in this group reported feeling lonely. However, instead of expressing a desire for social connections as the previous group did, participants in this group reported their deliberate choice to stay alone. One participant described the difference between the feeling of loneliness and the action of staying alone: *“There’s a difference between lonely and lonesome. Lonesome is when you really miss somebody, and lonely is by choice.” Interviewer: “And which one are you?” Participant: “By choice.”* Participants made this choice after having experienced other major stressors in life, making loneliness a secondary stressor. A participant reported feeling lonely, but when asked to recall the last time he felt lonely, he responded: *“None that I can remember, ‘cause I’ve been really pissed off about other shit.”* Loneliness did not create a dominant feeling of distress for participants in this group. Rather, they focused on creating conditions of social isolation, preferring to be alone. Participants described withdrawing from social interactions as a strategy for self-preservation: *“I feel more lonely when I’m around people than I am by myself. Because I don’t feel welcome a lot of times when I’m around too many people. Too much goin’ on, things that I don’t like. Stuff that I don’t want to really be around.”*

Compared to the mobility challenges, this group reported experiencing behavioral health issues and histories of trauma. Trauma was particularly pronounced for participants who experienced unsheltered homelessness, as one participant described: *“I’ve had guns pulled on me, I’ve had people try to break into – take over my tent, I’ve been held to gunpoint.”* Participants reported substance use as a maladaptive coping mechanism for trauma and physical pain, as almost all in this group reported historic or current substance use, particularly cocaine use. Two participants had suicide attempts or ideation. In the case of one participant, the intersection of mental health, substance use, and suicidality led to housing disruptions, which then exacerbated stress and the experience of loneliness: *“My drug addiction and my depression, I tried to commit suicide, and the owner of the hotel didn’t want me stayin’ there ‘cause she didn’t want to find me dead so she asked me to leave. And I left.”*

Participants reported a sense of abandonment by the healthcare system due to their lack of prescribed pain medications: *“It aint nothin’ that you could help me with, just like this pain that I go through. It get me pissed off about – but I internalize it because ain’t nothin’ nobody could do for me. So – I believe you have a lot of problems. I know you have some problems. So why would I put the problems out there.”* The significant frustration and anger felt by this participant led to his further resignation about institutional relationships helping him: *“f– the world, and you know what I’m sayin’, ‘cause just like – okay, they don’t care about me, why should I care about them? They know – you know I’m in here hurtin’, I can’t even get out the bed, 60-year-old man and I can’t get out of bed, been active my whole life but now you just toss me aside.”*

### Lonely – transient: acceptance and grief

Participants in this group described loneliness as less disturbing; instead, it felt transient and situational. Participants accepted loneliness as a part of the wide range of life experiences, partly due to aging and maturity: *“I used to feel lonely a lot when I had more people in my life – it’s not because of the amount of people I have in my life, I think it’s because of my age, maturity level, life experiences, where – feeling lonely is not so manifest in my life.”* One participant embraced the peacefulness of solitude after having experienced chronic homelessness. Interviewer: *“So it sounds like you spend most of the time by yourself?”* Participant: *“I don’t think I do it out of anxiety. It’s just that after over 20 years of homeless, it was compelling for me to sit by myself for a period of time just to get a sense of being inside.”*

When feeling lonely, participants in this group reported constructive coping behaviors, such as calling family members, going to a movie, or attending mutual aid groups. Some participants used humor or personal wisdom to help alleviate loneliness. When asked about what they do when feeling lonely, one participant said: *“I don’t consciously combat loneliness. What happens is, if I think, then I cannot sleep. The first thing I like to do is fix a cup of hibiscus tea. Then I ruminate. Then I make the mistake of ruminating over things I can’t fix.” (laughter).*

Participants in this group discussed loss of relationships as a major challenge in their current lives. Participants have commented that “*people have moved on”* or *“no one is nearby anymore.”* They reported grief and depression. A participant who recently lost his son described: *“The only thing that bothered me was, my son passed in November, so I mean, and it’s not really a bad deal, ‘cause off and on, it all depends – sometimes I think about him and I smile, think about things we have done, think about him from a little kid on, and then I guess when I’m really missin’ him, that kind of depresses me.”* Feelings of loneliness arose when past relationship losses triggered grieving, as one participant described: *“just sometimes it hits me. You know, I miss people. Everybody needs somebody to hold them sometimes, or like that. That’s the kind of lonely I be.”*

In contrast to their past relationships, participants’ current social relationships were generally instrumental, focusing on tangible supports rather than emotional connection, which furthered their longing for emotional connection – *“I got a lot of friends but I don’t have no friends. I’m really a loner. And I hang out with people, but not the way – I miss ‘em, like that. If I see ‘em, I see ‘em, if I don’t, I don’t.”*

### Not lonely – stability and connection

Participants in this group did not report being lonely. They seemed to have an established network to call upon when they felt alone, or they enjoyed being alone. Interviewer: *“Are there ever times when you feel lonely at all”?* Participant: *“No.”* Interviewer: *“No, you don’t feel lonely because you have people around you that you …”* Participant: *“Yeah, I have quite a few friends.”*

Participants in the not lonely group did not report issues with mobility, although they reported taking precautions to avoid falling. In general, participants in this group were able to move with some assistance. One participant who usually relied on mobility scooters said: *“If there’s no scooter, then [I] turn back around... I’m not going.”* Only one participant discussed mental health issues, who had been taking medication for bipolar disorder for 10 years and had recently discontinued medication.

In contrast to the other groups, participants in this group generally had more established and expansive social networks of family, friends, and service providers. Many participants reported high-quality relationships that had lasted for decades. More importantly, their social supports provided a combination of transactional support and non-transactional companionship, as shown in the following conversation with one participant. Interviewer: *“But right now you don’t feel lonely at all”*. Participant: *“No, man, we talk – we’ll talk two or three hours, I have to plug the phone [in to charge it], I have to keep talkin’.”*

Compared to the first three groups, those in the not lonely group were generally in more stable housing situations, although the quality of housing was still suboptimal. As one participant described the lack of basic amenities and safety concerns: *“And there’s no elevator in the building. In the neighborhood, I pass by, you hear gunshots at all hours”*.

## Discussion

Through a content analysis of loneliness among twenty older adults who have experienced homelessness, our study captured four types of loneliness experience, ranging from distressing loneliness to not lonely, and explored the distinct conditions that shaped each type of loneliness experience. Our study represents one of the few studies examining the subjective experience of loneliness among older adults who have experienced homelessness and offers knowledge to inform the development of interventions and programs that mitigate loneliness among older adults with intersecting vulnerabilities.

Our findings revealed the complex and heterogenous nature of loneliness in this population. Loneliness in our analysis varied by its severity and chronicity, and participants’ meaning-making about loneliness differed based on their personal experiences and needs. For example, participants who struggled with physical impairment understood loneliness differently from those who experienced violence, trauma, and more pronounced social marginalization. Participants in the “lonely–transient” group largely mentioned their longing for emotional companionship rather than more tangible, material supports. This finding resonates with emerging research conceptualizing loneliness as a multi-dimensional construct that is relative to individual contexts [16, 17, 18].

We found that loneliness and social isolation were not connected. While the literature recognizes that loneliness and social isolation are distinct phenomena (i.e., a subjective experience versus an objective state), research has posited that loneliness motivates people to seek and maintain social connections [19, 20]. Our findings highlighted an exception to this conceptualization. In our second group, “lonely – rather be isolated”, persistent trauma and social marginalization faced by participants, and exacerbated by experiences of homelessness, led to resignation from multiple social and institutional relationships despite the presence of loneliness. This observation in our finding supports previous research advocating for consideration of the social and structural barriers to reducing social isolation [11, 21, 22, 23, 24]. For example, restrictive policies prohibited homeless individuals from receiving housing respite from family and friends as it would threaten their eligibility for housing and social welfare assistance [23]. Individuals experiencing homelessness may choose self-isolation due to fear of unsafe living conditions and histories of trauma [24]. Our findings expand this line of research by linking structural barriers to perceived social isolation reflected by participants’ ambivalent feeling toward loneliness (i.e., rather be isolated). This is consistent with prior research suggesting that, in order to develop effective interventions addressing chronic loneliness, research needs to attend to the specific factors creating persistent and burdensome vulnerability for certain populations [15].

Our findings showed that the most distressing loneliness experience occurred among those with physical impairment and mobility challenges (i.e., “lonely – distressed”). This finding is consistent with prior research focusing on aging-related physical factors as contributors of loneliness, [25] suggesting a commonality in the mechanism of loneliness between older adults who have experienced homelessness and those who have not. Many of these mobility challenges were exacerbated by the lack of adequate healthcare, which is experienced by individuals experiencing homelessness, suggesting another structural and systematic level barrier to reducing loneliness.

We present several implications for future research on loneliness among marginalized populations, particularly those who have experienced homelessness. First, future research would benefit from a more comprehensive loneliness measurement to capture the heterogeneous nature of loneliness. An enhanced loneliness scale should be sensitive to the specific cultural norms and needs, especially for marginalized populations, to help identify nuanced variations in loneliness experience that may not align with existing metrics. Secondly, our study, along with prior research, [11, 21, 23, 24] highlighted the distinct social and structural barriers to reducing loneliness, such as interpersonal violence and structural violence while experiencing homelessness. Future research should continue to explore the mechanisms through which social and structural barriers play a role in loneliness. One such mechanism could be stress, as research has suggested a mediational role of stress between life course adversities and loneliness [26, 27]. Finally, rather than exclusively targeting individual-level factors, such as social skills, [28] interventions should place greater emphasis on addressing the social and structural barriers that perpetuate loneliness. This shift in approach could lead to interventions and programs that are better aligned with the unique needs and challenges faced by marginalized populations, including older adults with a history of homelessness.

Our findings yielded several policy and practical implications. First, while the causal relationship between the built environment and social support is nuanced in prior research, [22] our findings illustrated that providing stable and safe housing is a foundational step in alleviating loneliness. Policies should prioritize housing and supportive services that specifically target residents’ health and well-being outcomes such as loneliness. Individuals experiencing homelessness are considered older at a younger age (i.e., 50 years old), therefore policies should attend to architecture, the built environment, programming, and other services (e.g., transportation) to optimize access to and utilization of formal social support and services for people experiencing loneliness. Given the prevalence of trauma-related factors in shaping loneliness experience, social service organizations and providers should prioritize trauma-informed care when working with older adults who have experienced loneliness. This approach involves recognizing the potential trauma history of individuals and providing supports that are responsive to their life experiences. Finally, the complex nature of loneliness calls for an interdisciplinary approach in care for older adults experiencing or at risk for loneliness. Healthcare providers, social workers, housing agencies, and urban space designers should combine expertise from various fields to facilitate a more holistic and person-centered care system that facilitate adequate access to social support and resources.

Limitations of our study included a relatively small sample size, which resulted in a small number of participants in each loneliness group. A larger sample size could have allowed for a more systematic evaluation of the distinct conditions of loneliness. We collected data at one point in time, so we were unable to examine potential longitudinal changes in loneliness, especially before and after changes in housing status. We collected data before the COVID-19 pandemic, which had huge impacts on loneliness [29]. We were unable to capture the post-pandemic loneliness experience among our participants.

## Conclusion

This qualitative study used a content analysis approach to examine loneliness experience among older adults who have experienced homelessness. We found four types of loneliness experiences and the distinct individual, social, and structural conditions of each loneliness experience. Our findings highlighted the complex and heterogenous nature of loneliness and underscored the importance of the social and structural barriers to alleviating loneliness. Our findings provided several research, policy, and practical implications in better addressing loneliness among older adults with intersecting vulnerabilities. We called for a trauma-informed, holistic approach in providing healthcare and social support services.

## Data Availability

The datasets generated and analyzed during the current study are not publicly available but are available from the corresponding author on reasonable request.
